# A Comprehensive Analysis on Wearable Acceleration Sensors in Human Activity Recognition

**DOI:** 10.3390/s17030529

**Published:** 2017-03-07

**Authors:** Majid Janidarmian, Atena Roshan Fekr, Katarzyna Radecka, Zeljko Zilic

**Affiliations:** Electrical and Computer Engineering Department, McGill University, Montréal, QC H3A 0E9, Canada; atena.roshanfekr@mail.mcgill.ca (A.R.F.); katarzyna.radecka@mcgill.ca (K.R.); zeljko.zilic@mcgill.ca (Z.Z.)

**Keywords:** human activity recognition, machine learning, supervised classification, wearable sensors, sensors heterogeneities

## Abstract

Sensor-based motion recognition integrates the emerging area of wearable sensors with novel machine learning techniques to make sense of low-level sensor data and provide rich contextual information in a real-life application. Although Human Activity Recognition (HAR) problem has been drawing the attention of researchers, it is still a subject of much debate due to the diverse nature of human activities and their tracking methods. Finding the best predictive model in this problem while considering different sources of heterogeneities can be very difficult to analyze theoretically, which stresses the need of an experimental study. Therefore, in this paper, we first create the most complete dataset, focusing on accelerometer sensors, with various sources of heterogeneities. We then conduct an extensive analysis on feature representations and classification techniques (the most comprehensive comparison yet with 293 classifiers) for activity recognition. Principal component analysis is applied to reduce the feature vector dimension while keeping essential information. The average classification accuracy of eight sensor positions is reported to be 96.44% ± 1.62% with 10-fold evaluation, whereas accuracy of 79.92% ± 9.68% is reached in the subject-independent evaluation. This study presents significant evidence that we can build predictive models for HAR problem under more realistic conditions, and still achieve highly accurate results.

## 1. Introduction

The maturity of pervasive sensing, wireless technology, and data processing techniques enables us to provide an effective solution for continuous monitoring and promote individual’s health. Today, the miniature sensors can be unobtrusively attached to the body or can be part of clothing items to observe people’s lifestyle and behavior changes [1]. According to study presented in [2], on-body sensing proves to be the most prevalent monitoring technology for the gait assessment, fall detection and activity recognition/classification. As such, extensive research has been undertaken to select or develop reasoning algorithms to infer activities from the wearable sensor data. Human activity recognition thattargets the automatic detection of people activities, is one of the most promising research topics in different areas such as ubiquitous computing and ambient assistive living [3]. Low-cost, yet highly reliable accelerometer is the most broadly used wearable sensor for the sake of activity recognition and could provide high classification accuracy of 92.25% [4], 96% [5], and 99.4% [6]. 3-D accelerations can be represented as:

$$\vec{A}=\frac{d\vec{v}}{dt}=\left(\vec{g}+\vec{l}\right),\;\left(\begin{array}{c} \begin{array}{c} A_{x}\\ A_{y} \end{array}\\ A_{z} \end{array}\right)=\left(\begin{array}{c} \begin{array}{c} g_{x}+l_{x}\\ g_{y}+l_{y} \end{array}\\ g_{z}+l_{z} \end{array}\right)$$

where $\vec{A}$ (acceleration), $\vec{g}$ (acceleration due to gravity) and $\vec{l}$ (applied linear acceleration) are measured in $\frac{m}{s^{2}}$. There is a large amount of work on the use of sensing for activity monitoring and behavior profiling. For example, there are surveys [7,8,9] that provide an outline of relevant research and applicable techniques. In the real-life scenarios, the performance of a recognition system is often significantly lower than in reported research results. Igual et al. recently applied two different fall detection algorithms to show that the performances of the fall techniques are adversely affected with cross-dataset evaluation [10]. It shows that the performance of a fall detector reduced when it is tested on a dataset different from the one used for training. This is because there exist variations in training and testing device hardware, sensor models, and their operating system characteristics among others [11]. The performance of recognition models mainly depends on the activity set, training data quality, extracted features, and learning algorithms. Since each Machine Learning (ML) model in the literature was trained with a specific dataset and activity set, there is no significant evidence to claim that any predictive model is more precise than the others. In other words, the classification model is built based on the collected samples under specific conditions involving sensor type, position and orientation of sensors on the human body, sampling rate, and activity performance style. Therefore, the trained model may not be directly applied to other related datasets and may fail to understand the pattern, if there is any change in the sensor characteristics, data acquisition scenarios or the users (concerning, e.g., age, weight or physical fitness). For instance, different accelerometer sensors often suffer from various biases and thus differ in precision and resolution. This issue, combined with sampling rate instability of each device introduces major challenges for the HAR system design [12]. The difference in the styles of performance of an activity also poses some challenges for application developers and researchers. Stisen et al. [11] showed that even OS type and CPU load have also drastic negative effects on recognition accuracy. Therefore, we aim to comprehensively evaluate the machine learning algorithms to extend the applicability of the trained model dealing with diverse accelerometer measurements. In this work, we have aggregated 14 well-known benchmark datasets that are publicly available to the research community and, in each dataset, data have been collected with different devices (commercial mass-marketed or research-only devices), acquisition protocols (under naturalistic circumstances or laboratory environments), participants, sensors placements, models and biases, motion artifacts and sampling rate heterogeneities to have a big realistic dataset. Considering these challenges makes it difficult to obtain a robust activity recognizer that is invariant to biases and performs well on unseen datasets. This is the first time to the best of our knowledge that such rigorous activity recognition evaluation at a large-scale on ML techniques is investigated. This study will explain the pros and cons of variety of learning methods and will speed up implementation of robust recognition algorithms using wearable accelerometer sensors. We report the effects of heterogeneities on various classifiers considering two cross-validation techniques. K-fold (*k* = 10) is the most widely accepted methodology to compute the accuracy of a developed model in HAR problem [13]. In this technique, the model is trained using *k* – 1 of the folds as training data and the obtained model is validated on the remaining part of the data to compute the accuracy or other performance metrics. However, to explore the limitations of finding a personalization approach caused by large variance in per-user accuracy, a subject-independent cross-validation technique, Leave-One-Subject-Out (LOSO), is also considered.

This paper in organized as follows. In Section 2, backgrounds in the field of human activity recognition and the adopted methodologies including feature extraction/selection and classification techniques with the parameters of prediction functions are addressed. The used datasets in this study will be discussed and listed in Section 3. Section 4 presents the experimental results obtained with different cross-validation techniques. Finally, a conclusion and some research perspectives are given in Section 5.

## 2. Backgrounds and Methodologies

**Figure 1:**
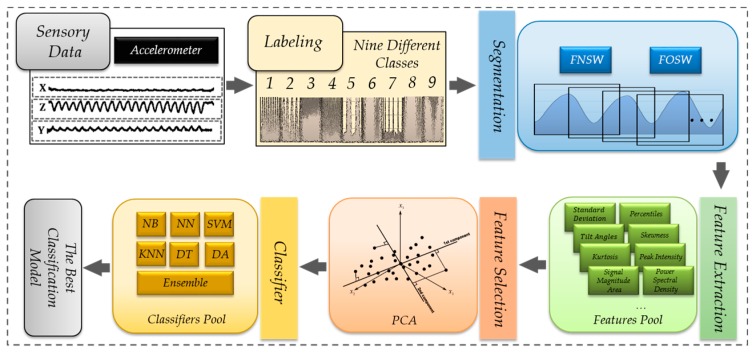
Sensor-based activity recognition procedure.

Human Activity Recognition (HAR) starts with collecting data from the motion sensors. The data are partitioned into windows to apply feature extraction thereby filtering relevant information in the raw signals. Afterward, extracted features are used as inputs of each classifier that ultimately yields the HAR model. To evaluate the effect of sensing heterogeneity on classifiers, we do not perform any preprocessing steps. This problem is formulated as follows:

Definition: With *p* extracted features from the motion sensors, given a set $W=\left\{w_{1},w_{2},\ldots ,w_{n}\right\}$ of labeled and equal-sized time windows, and a set $A=\left\{a_{1},a_{2},\ldots ,a_{l}\right\}$ of activity labels, the goal is to find the best classifier model *C*, such that for any $w_{k}$ which contains a feature set $F_{k}=\left\{f_{k,1},f_{k,2},\ldots ,f_{k,p}\right\}$, the predicted label ${\hat{a}}_{k}=C\left(F_{k}\right)$ is as identical as possible to the actual activity performed during $w_{k}$. *p* is the number of features in vector $F_{k}$ extracted from $w_{k}$. Figure 1 depicts the whole system flow of sensor-based activity recognition for nine activities.

### 2.1. Data Segmentation, Feature Extraction and Selection

Stream of sensory data needs to be divided into subsequent segments. Fixed-size Sliding Window (FSW) is the most common method in segmentation step where the data stream is allotted into fixed-length windows with no inter-window gaps. If there is no degree of overlap between adjacent windows, it is called Fixed-size Non-overlapping Sliding Window (FNSW). The second method is Fixed-size Overlapping Sliding Window (FOSW), which is similar to FNSW except that the windows overlap during segmentation [8,9]. The use of overlap between adjacent windows has been shown to be effective in classification problem using wearable sensor data [14,15]. Finding the optimal window size *t* is an application-dependent task. The window size should be properly determined in such a way that each window is guaranteed to contain enough samples (at least one cycle of an activity) to differentiate similar movements. In addition, increasing the window size does not necessarily enhance the accuracy but may add computational complexity (causing higher latency). To better address the challenge, we analyze the influence of window sizes (ranging from 1 s to 15 s) on the classification performance.

Feature extraction is to obtain the important characteristics of a data and represent them into a feature vector used as input of a classier [16]. Table 1 gives details about the most effective time/frequency-domain and heuristic features in the literature in the context of activity recognition. Due to low computational cost and high discriminatory ability of time-domain features, they are the most frequently employed features for real-time applications. We compute all the features listed in Table 1 using each reading of accelerometer sensor consists of 3-D accelerations (x, y, z). However, to minimize the effects of sensor orientation, we add another dimension to the sensor readouts, which is called the magnitude of the accelerometer vector, i.e., $\sqrt{x^{2}+y^{2}+z^{2}}$, because it is less sensitive to the orientation changes [17]. It is worth noting that the correlation features are calculated between each pair of axes, and the tilt angles are estimated by combination of all three axes as shown in Table 1. Each classifier is fed with the feature vectors obtained from fusing data at the feature level. As a result of the above feature extraction process, a total of 176 features are obtained for each segment and then scaled into interval [0, 1] using min-max normalization so as to be used for classification.

**Figure 2:**
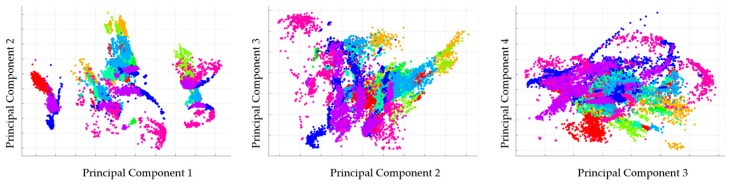
The pairwise scatter plots of the first four components.

As not all features are equally useful in discriminating between activities, Principal Component Analysis (PCA) is applied to map the original features $F_{k}=\left\{f_{k,1},f_{k,2},\ldots ,f_{k,p}\right\}$ into a lower dimensional subspace (i.e., new mutually uncorrelated features) ${F^{\prime }}_{k}=\left\{{f^{\prime }}_{k,1},{f^{\prime }}_{k,2},\ldots ,{f^{\prime }}_{k,m}\right\}$, where *m* ≤ *p* [18]. It also significantly reduces the computational effort of the classification process. The PCA components can be counted by *X* = *YP*, where *X* and *Y* are centering and input matrix, respectively and *P* is a matrix of eigenvector of the covariance vector matrix *C_x_* = *PΛP^T^*. Λ is a diagonal matrix whose diagonal elements are the eigenvalues corresponding to each eigenvector [19]. The new feature vectors are so-called principal components and arranged according to their variance (from largest to lowest). To keep the essential information in acceleration data that describe human activity, we take the first principal components that explain 95% of the total variance. The pairwise scatter plots of the first four components (transformed features) of one of test cases are given in Figure 2. As expected, the first components (the first component against the second component) for different classes are better clustered and more distinct.

### 2.2. Machine Learning Techniques

In this study, we are dealing with the supervised machine learning methods where the class labels are used to train the feature vectors extracted from each separate segment of data. We attempt the most complete analysis on performance of classifiers to discriminate among different types of activity. Wide range of machine learning methods have been applied for recognition of human activities such as decision tree (DT) [20,21,22,23,24,25], Support Vector Machines (SVM) [4,5,20,23,25,26,27,28], K-Nearest Neighbors (KNN) [20,21,23,25,27], Naïve Bayes (NB) [20,23,25,29], artificial Neural Network (NN) [23,24,26,30] and ensemble of classifiers [6,20,25,26].

We explore 293 different classifiers including Decision Tree, Discriminant Analysis, Support Vector Machines, K-Nearest Neighbors, Ensemble Methods, Naïve Bayes and Neural Network with their different parameters. The methods and their parameters setting are described and given IDs in Appendix A. The main objective of implementing different classification techniques is to review, compare and evaluate their performance considering the most well-known heterogeneous datasets publicly open to the research community. We are going to intertwine different issues and suggest solutions if we expect reasonable results in the practical applications.

## 3. Datasets

To design a robust learning model working in more realistic conditions, we combined 14 datasets, focusing on accelerometer sensors that contain several sources of heterogeneities such as measurement units, sampling rates and acquisition protocols that are present in most real-world applications. Table 2 listed the datasets and brought the details of the collected data in each project. In total, the aggregated dataset has about 35 million acceleration samples from 228 subjects (with age ranging from 19 to 83) of more than 70 different activities. This is the most complete, realistic, and transparent dataset in this context.

**Figure 3:**
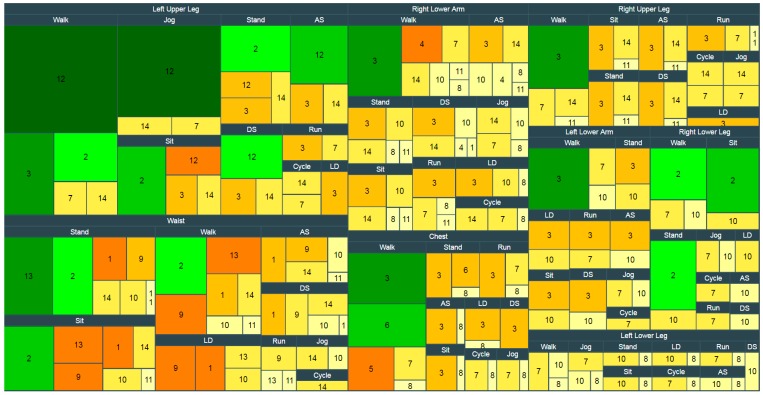
The rectangular tree map that presents dense volumes of data in a space filling layout to see datasets contributions in each target position. Laying Down (LD), Ascending Stairs (AS), Descending Stairs (DS). The number inside each rectangle indicates the dataset number (see first column of Table 2).

We considered 10 major positions on the body i.e., Waist (W), Right Lower Arm (RLA), Left Lower Arm (LLA), Right Upper Arm (RUA), Left Upper Arm (LUA), Right Lower Leg (RLL), Left Lower Leg (LLL), Right Upper Leg (RUL), Left Upper Leg (LUL), and Chest (C). All sensors positions described in each dataset have been mapped into the major positions. For example, if a subject puts the cellphone in the left front pants pocket, we consider it as Left Upper Leg (LUL) position, or wrist, which is a great place for many commercial wearables, is considered as RLA/LLA in this paper. In this field, numerous studies [31,32] have shown that the performance of HAR systems strongly depends on sensor placement since the number and the placement of inertial sensors have direct effects on the measurement of bodily motions. Each placement turns out to be more suitable in terms of performance for particular activities. Besides, having fewer sensors attached to the body is more preferable since wearing multiple ones can become burdensome and is not well-accepted. Therefore, we limited our modeling and analysis for single-accelerometer data while we still expect a sufficiently high recognition rate for the picked activities. According to the datasets, the most examined activities (top activities) are walking, running, jogging, cycling, standing, sitting, lying down, ascending and descending stairs which also represent the majority of everyday living activities. Another observation we find is that in eight major positions we have data for all top activities. Therefore, we choose them as target activities for eight separate positions (W, RLA, LLA, RUL, LUL, RLL, LLL and C). We created rectangular tree map that presents dense volumes of data in a space filling layout allowing for the visual comparison of datasets contributions in each target position (see Figure 3). For example, as depicted in Figure 3, datasets 3, 5, 6, 7 and 8 contribute data for constructing the chest dataset with nine activities.

## 4. Experimental Results and Discussions

**Figure 4:**
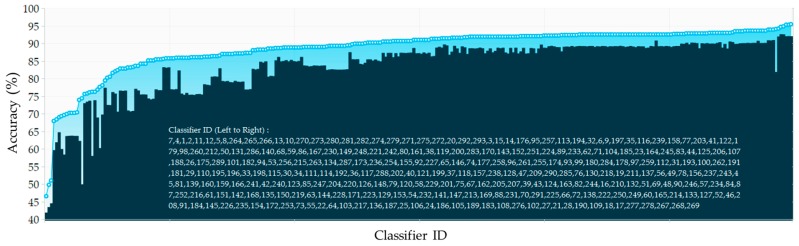
The minimum and maximum accuracy of each classifier over different window sizes, ranging from 1 s to 15 s, with the waist accelerometer data.

**Figure 5:**
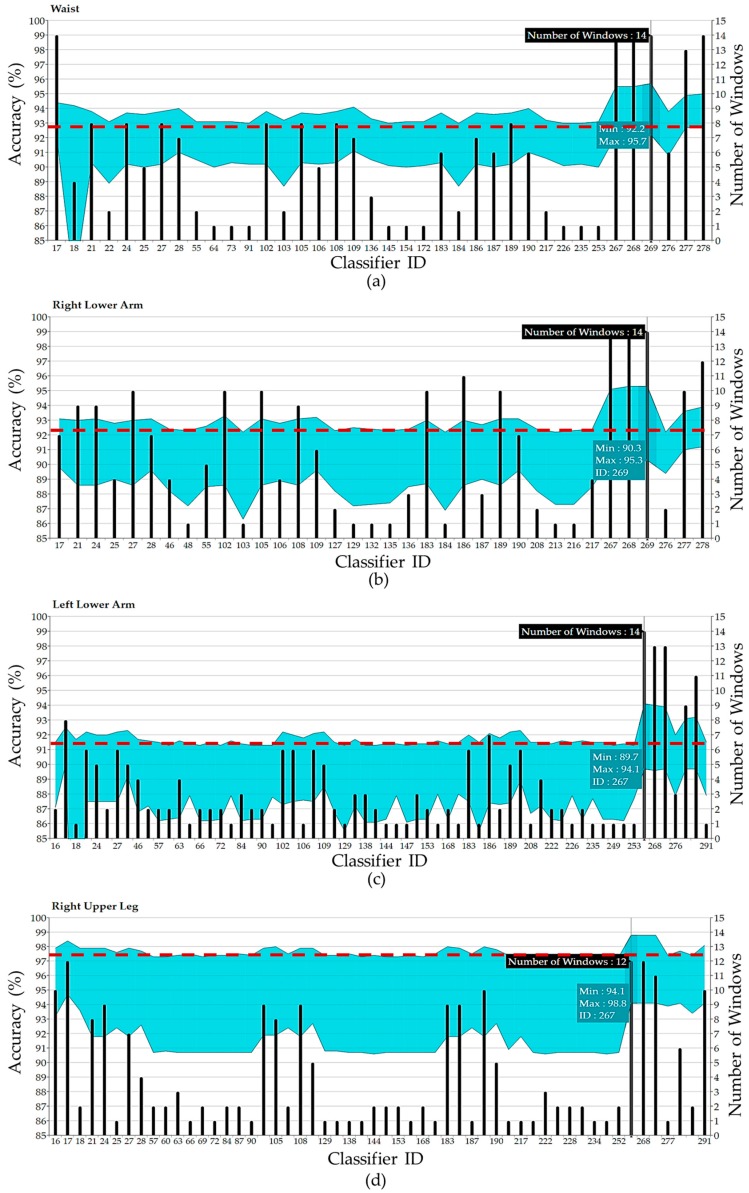

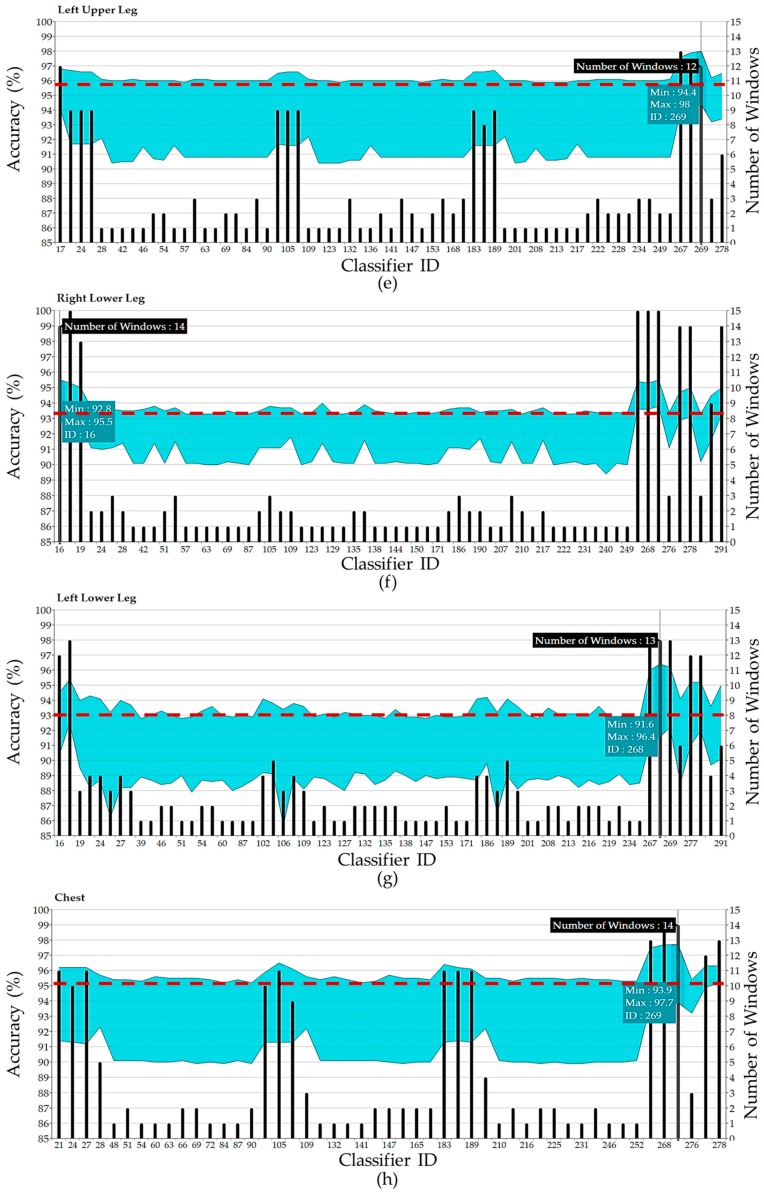
The range of *topClassifiers* accuracies for (**a**) Waist; (**b**) RLA; (**c**) LLA; (**d**) RUL; (**e**) LUL; (**f**) RLL; (**g**) LLL; and (**h**) Chest.

In this section, we report the effects of the heterogeneities, from sensors characteristics, data collection scenarios and subjects, on various feature representation techniques and 293 classifiers considering two cross-validation techniques. First, the 10-fold cross-validation strategy is applied as one of the most accurate approaches for model selection. Figure 4 shows the minimum and maximum obtained accuracy of each classifier over different window sizes with the waist accelerometer data. The algorithms are sorted according to the best obtained accuracy. Considering the best acquired accuracy for each classification category in this position, the ensemble methods KNN (Subspace) and Tree (Bagging) achieved the highest activity recognition rate whereas DT performed the worst. Further DA, DA (Subspace), Tree (AdaBoost), Tree (RUSBoost) and NB performed almost equal but worse than SVM, NN and KNN. As can be seen, some classification learning algorithms are more sensitive to parameters settings and window size and may thus be more likely to exhibit significant differences. To have a better and deeper investigation, we extracted the classifiers with top 5% accuracies and call them “*topClassifiers*” for each position. Figure 5 depicts the range of *topClassifiers* accuracies for each position. A red dashed line annotation shows the 95th percentile of obtained accuracies.

**Figure 6:**
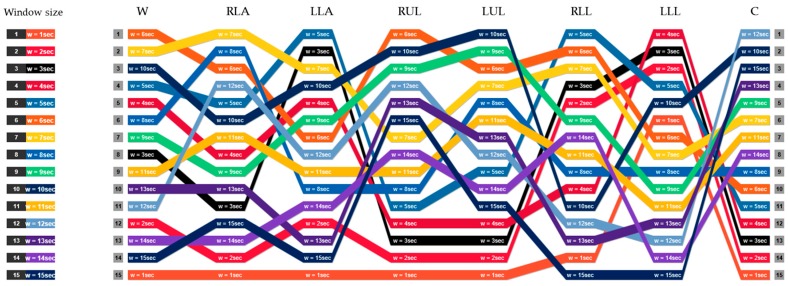
The rank of window sizes in providing the best accuracy in each position.

As can be seen in this figure, most of the recognition methods remain consistent in their relative performance across different accelerometer data obtained from different positions. As explained in Section 2, finding the optimal length of window size is an application-dependent task. The window size should be properly determined in such a way that each window is guaranteed to contain enough samples to differentiate similar activities or movements. Thus, we consider different window sizes ranging from 1 s to 15 s in steps of 1 s to ensure the statistical significance of the calculated features. It comprises most of the values used in the previous activity recognition systems [8]. Figure 6 describes the ranking of different window sizes in providing the best accuracy (among all classifiers) in each position. For example, window of length 7 s provides the best classification accuracy when the sensor is attached on the RLA. The second best accuracy value for RLA is achieved with window size 8 s. The first line in this figure shows the window sizes of the best accuracies for each position. In contrast to LLL where the top four accuracy values have been observed in small window sizes ranging from 2 s to 5 s, chest provides the top-rank accuracy values in large window sizes from 10 s to 15 s. This observation is more highlighted in orange line (w = 1 s) where all positions obtain the worst case accuracy values except for the LLL. However, in some cases we can change the window size (increase/decrease) at the expense of a subtle performance drop. For example, in chest position, the window size can be reduced from 15 s to 7 s by only tolerating 0.16% in recognition performance.

**Figure 7:**
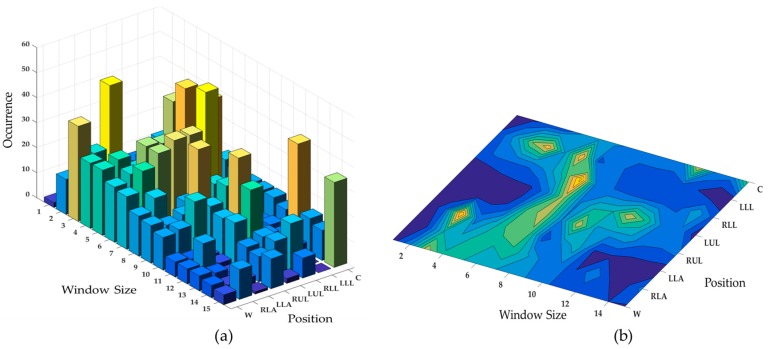
Effect of window size to gain meaningful information for the activity classification in (**a**) 3D and (**b**) 2D representations.

The bar charts in Figure 5 indicate the number of window sizes (1 s to 15 s) in which the *topClassifiers* provided good results (top 5%). An interesting observation from the bars is that some classifiers such as KNN (Subspace), Tree (Bagging) and SVM work well with most windows sizes. This means they could mitigate the effect of window size to gain meaningful information for the activity classification process. To have a better understanding of window size effect on accuracy, Figure 7 shows the *topClassifiers* across all window sizes in each position. As can be observed, the interval 3–10 s proves to provide the best accuracies in most cases considering the target activities. This range can be reduced if fusion of multiple sensors is used for feature extraction. Another point worth mentioning that is for the underlying periodic activities, large window sizes do not necessarily translate into a better recognition performance.

**Figure 8:**
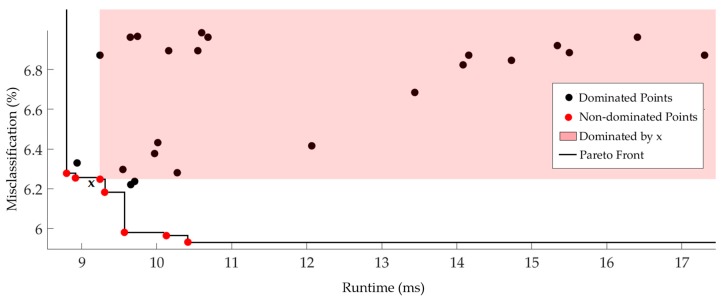
Illustration of some Pareto fronts when minimizing two objectives (misclassification and classification runtime) according to the obtained results in the waist.

**Figure 9:**
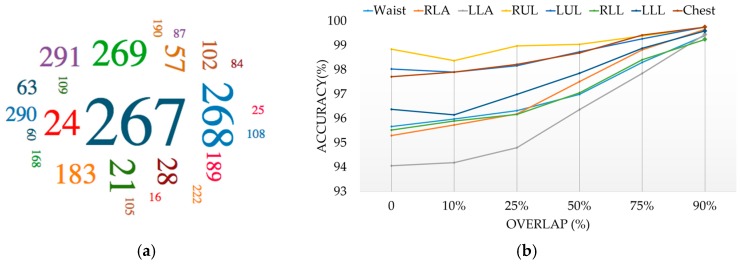
(**a**) Overall view of the non-dominated classifiers (classifier ID) and their power in providing high recognition accuracy (**b**) recognition system capabilities for diverse overlap values.

Although accuracy is necessary for all recognition algorithms, it is not the only parameter to consider in designing a recognition model. The runtime complexity (classification step) is another important challenge as the model should be working fast and responsive regardless of where it is deployed i.e., on/off-device. Thus, we make use of the concept of Pareto optimality to extract superior solutions from *topClassifiers* to tradeoff classifier accuracy and runtime. We consider two objective functions, i.e., misclassification and runtime, to be minimized. A feasible solution *x* dominates a feasible solution *y* when

$$\forall \;i,f_{i}\left(x\right)\leq f_{i}\left(y\right)$$

where $f_{i}$ is the *i*th objective function. However, in many problems, there is usually no single solution that is superior to all others, so the non-dominated solutions compose the Pareto front. For example, in Figure 8, we populate the runtime-accuracy plane with some *topCalssifiers* for waist position and depict the Pareto front. The shaded area represents the region in $f_{1}\times f_{2}$ space that is dominated by the point *x* which is non-dominated and hence belong to the Pareto front [38]. All points in this region are inferior to *x* in both objectives. In addition, if we want to minimize an objective to a constraint, e.g., $f_{1}\left(x\right)<c$, the Pareto front provides the solution for all possible values of the cap *c* [38]. Therefore, the Pareto front contains significantly richer information than one obtains from single-objective formulations. Table 3 summarizes the non-dominated classifiers in each position. The results show a clear tradeoff between classifiers runtime and accuracy. There is no strong relation between the sensor position and classification performance. In overall, the highest classification accuracy was achieved by KNN (Subspace), and KNN stayed in the second place, which is followed by SVM and NN. Figure 9a depicts the overall view of the non-dominated classifiers and their power in providing high recognition accuracy. The size of each classifier ID in this figure represents the number of times that the corresponding classifier has been reported in Table 3.

The KNN has the best classification runtime (7 ± 1.78 ms) fed with a feature vector among all of them. While for classification accuracy, it is always after its ensemble method KNN (Subspace). In all cases, KNN (Subspace) with average accuracy (96.42% ± 1.63%) provided better results than all other non-dominated classifiers, with the exception of data from the RLL, where the SVM (95.52%) provided superior accuracy. However, SVM’s prominence is negligible while considering its runtime (113.05 ms) and no significant accuracy improvement (0.1%). Given the accuracy results stated in Table 3, although NN classifiers provide promising results in most cases, they are dominated by other techniques and could be only among the selected methods in three positions RUL, RLL and LLL. With a closer look at the classifications results in Figure 5 and tabulated results, we find out ensemble method Tree (Bagging) is a very strong method and is among *topClassifiers* in all cases, but is always outperformed by other methods in terms of both accuracy and runtime. According to the selected KNN classifiers, the distance method affects most in the performance. City block and Euclidean have been the best choices and too large value for *k* does not improve the performance as it destroys locality. We also can draw another conclusion that there was no significant difference in KNN (Subspace) performance with different number of learners (i.e., 10, 20 and 40). Therefore, applying fewer learners is preferentially utilized due to its much lower runtime.

Regarding the position analysis, the best performance (98.85% and 98.03%) is achieved with the aggregated data on RUL and LUL. Chest is the next best performing placement (97.72%) compared to the RLL (95.52%), LLL (96.38%), RLA (95.30%), LLA (94.06%) and Waist (95.67%).

**Figure 10:**
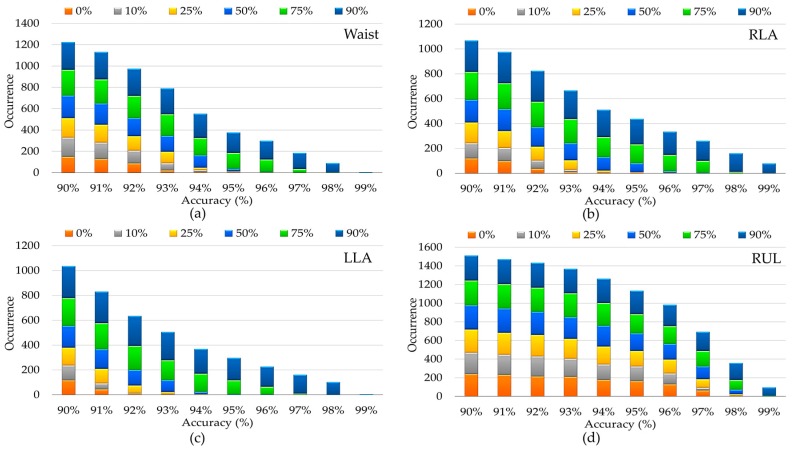

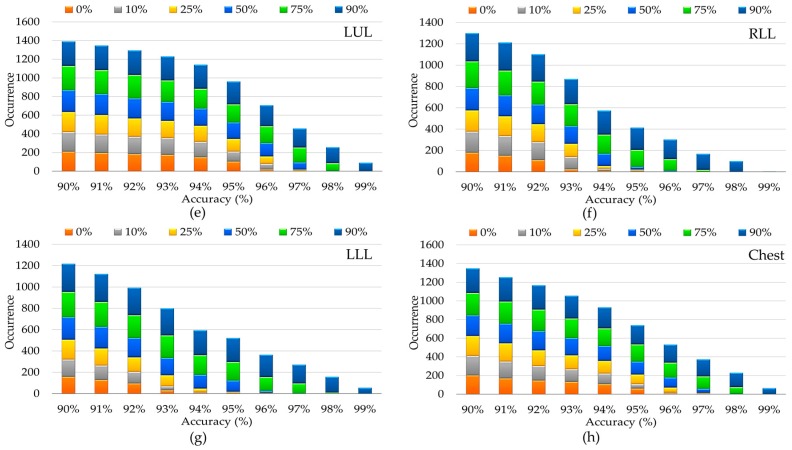
Analysis of number of classifiers, which provide good results (90%–99%) by taking different overlap sizes into account for different positions.

Different studies [15,26,39] show that the use of overlap between successive sliding windows help the classifiers to be trained with more feature vectors and consequently improve the recognition performance. However, it adds more computational work needed to process the overlapped data multiple times. To evaluate the effectiveness of the degree of overlap, the overlaps of 10%, 25%, 50%, 75% and 90% are used where the percentage is the amount the window slides over the previous window. For instance, a sliding window with 25% overlap will start the next window while the previous window is 75% complete. The value can range from 0% to 99%, since a 100% sliding window is erroneous. Figure 9b illustrates the recognition system capabilities for diverse overlap values while keeping the best window size in each position (see Figure 6). The results demonstrate that the performance tendency is increased in most cases by overlapping more data segmentations. An average increase of 3.28% in the best accuracy was found between the 0% and 90% overlap scenarios and all obtained with ensemble of KNN. Figure 10 also illustrates the number of classifiers that provide good results (90%–99%) by considering different overlap sizes. The larger the overlap, the more improvement is expected in performance. As described, this is because more features can be trained and consequently the predictive model almost certainly works better in testing phase; however, it suffers from more training time.

In activity recognition problem, K-fold cross-validation is an accurate approach for model selection; however, Leave-One-Subject-Out (LOSO), which is also called subject-independent cross-validation, can be used to avoid possible overfitting and is one of the best approaches in estimating realistic performance. Because LOSO reflects inter-subject variability and tests on yet-unseen data, it consequently leads to a decrease in accuracy. According to combined datasets in each position (see Figure 3), we conducted LOSO evaluation where the KNN (Subspace) is trained on activity data for all subjects except one. Then, the classifier is tested on the data for only the subject left out of the training data set. The procedure is then repeated for all subjects in each position and the mean accuracy is reported. For each position, the window size and overlap are set based on the best acquired results from 10-fold evaluation. Based on the aggregated data for each position, the models trained in LUL (92.35%) and LLL (90.03%) positions were not affected much by interpersonal differences in the body movement of subjects and could obtain relatively good results of accuracy. They are followed by Chest (86.31%) and RLL (83.51%), which are better performing placements compared to the lower arm positions. The RLA (64.62%) was influenced the most and LLA (77.12%) accounted for a substantial decrease in accuracy, as well. Result of Waist (72.53%) was very similar to one reached by RUL (72.91%), but both suffer performance degradation by more than 25%. As expected the accuracy of recognition in all positions was reduced since the trained model deals with a set of data that might have new measurement characteristics. As the best classifier of each position in average goes down 17.13% in accuracy, there is a need for further studies investigating new relevant features and novel machine learning model to be sufficiently flexible in dealing with inter-person differences in the activities’ performances in a position-aware scenario.

## 5. Conclusions

In this study, different machine learning techniques were deeply explored when heterogeneity of devices and their usage scenarios are intrinsic. In addition, each position was analyzed based on the aggregated tri-axial accelerometer data from different datasets. Hence, the quantitative comparison of the classifiers was hindered by the fact that each position is explored with a different aggregated dataset. In each position investigation, in addition to different sources of heterogeneities in data, there are also different factors such as body shape, clothing, straps, belt and accidental misplacements/disorientations (in the form of rotations or translations) that make the analysis harder to have a solid model. The averaged results showed 96.44% ± 1.62% activity recognition accuracy when using K-fold and 79.92% ± 9.68% accuracy when using a subject-independent cross-validation. According to the obtained results, it is clear that new data with different sources of heterogeneities could significantly reduce the accuracy with more than 32% (e.g., in RLA) based on LOSO evaluation. An overall look into the results, KNN and its ensemble methods showed stable results over different positions and window sizes, indicating its ability in designing a robust and responsive machine learning model in the wearables, and they are followed by NN and SVM. However, as we showed in this paper, the choice of parameter values in each classifier can have a significant impact on recognition accuracy and should be taken into account (see Appendix A). Considering the promising results in this pilot study, we intend to work on novel features extraction methods and classifiers that outperform classical classification methods while better dealing with inter-person differences and data diversities. Another point that deserves to be further assessed is optimizing the runtime performance since it has a great role in efficiency of the cloud-based machine learning deployments.

## Figures and Tables

**Table 1:** The features list.

Feature	Description	Feature	Description
***Mean***	$\mu_{s}=\frac{1}{n}\sum_{i=1}^{n}s_{i}$	***Skewness***	$\frac{1}{n{\sigma_{s}}^{3}}\sum_{i=1}^{n}{\left(s_{i}-\mu_{s}\right)}^{3}$
***Minimum***	$min\left(s_{1},s_{2},\ldots s_{n}\right)$	***Kurtosis***	$\frac{1}{n{\sigma_{s}}^{4}}\sum_{i=1}^{n}{\left(s_{i}-\mu_{s}\right)}^{4}$
***Maximum***	$max\left(s_{1},s_{2},\ldots s_{n}\right)$	***Signal Power***	$\sum_{i=1}^{n}s_{i}^{2}$
***Median***	$median\;\left(s_{1},s_{2},\ldots s_{n}\right)$	***Root Mean Square***	$\sqrt{\frac{1}{n}\sum_{i=1}^{n}{s_{i}}^{2}}$
***Standard Deviation***	$\sigma_{s}=\sqrt{\frac{1}{n}\sum_{i=1}^{n}{\left(s_{i}-\mu_{s}\right)}^{2}}$	***Peak Intensity***	*The number of signal peaks within a certain period of time*
***Coefficients of Variation***	$\frac{\sigma_{s}}{\mu_{s}}$	***Pearson's Correlation Coefficient***	$\frac{cov\left(a,b\right)}{\sigma_{a}\sigma_{b}}$
***Peak-to-peak Amplitude***	max (s)−min(s)	***Inter-axis Cross-Correlation***	$\frac{\sum_{i=1}^{n}\left(a_{i}-\mu_{a}\right)\left(b_{i}-\mu_{b}\right)}{\sqrt{\sum_{i=1}^{n}{\left(a_{i}-\mu_{a}\right)}^{2}\sum_{i=1}^{n}{\left(b_{i}-\mu_{b}\right)}^{2}}}$
***Percentiles***	$t=\frac{np_{i}}{100}+0.5,\;p_{i}=10,\;25,\;50,\;75,\;90$	***Autocorrelation***	$R\left(k\right)=\frac{1}{\left(n-k\right){\sigma_{s}}^{2}}\sum_{i=1}^{n-k}\left(s_{i}-\mu \right)\left(s_{i+k}-\mu \right)$ ∀ k<n; *the height of the first and second peaks and the position of the second peak of* R(k)
	$percentile\left(s,p_{i}\right)=\left(1-f\right)s_{k}+fs_{k+1}$		
	*k = integer part of t; f = fractional part of t*		
***Interquartile Range***	percentile(s,75 )− percentile(s,25 )	***Trapezoidal Numerical Integration***	$\int_{1}^{n}s\left(x\right)dx$ *using Multiple Segment Trapezoidal Rule*
***Pitch Angle***	$arctan\left(\frac{x_{i}}{\sqrt{y^{2}+{z_{i}}^{2}}}\right)$	***Signal Magnitude Area***	$\frac{1}{n}\sum_{i=1}^{n}(|x_{i}\left|+|y_{i}\right|+|z_{i}|)$
***Roll Angle***	$arctan\left(\frac{y_{i}}{\sqrt{x^{2}+{z_{i}}^{2}}}\right)$	***Signal Vector Magnitude***	$\frac{1}{n}\sum_{i=1}^{n}\sqrt{{x_{i}}^{2}+y^{2}+{z_{i}}^{2}}$
***Median Crossings***	t = s − median(s)	***Power Spectral Density***	$\frac{1}{n}\sum_{i=1}^{n-1}{\left(s_{i}\cos\frac{2\pi fi}{n}\right)}^{2}+{\left(s_{i}\sin\frac{2\pi fi}{n}\right)}^{2}$ *f denotes the f^th^ Fourier coefficient in the frequency domain; the positions and power levels of highest 6 peaks of PSD computed over the sliding window; total power in 5 adjacent and pre-defined frequency bands.*
	$MC=\overset{n}{\underset{i=1}{\sum }}sgn(t_{i}.t_{i+1})$		
	*sgn(a,b) = {1 if (a.b) < 0; 0 if (a.b) > 0}*		

**Table 2:** The datasets used in this study.

Dataset	Number of Subjects	Sensor Type	Frequency	Sensor Placement	Activity Type	Description
(1) [5]	30 (19–48 year)	accelerometer gyroscope (Samsung Galaxy S II smartphone)	50 Hz	waist (1)	walking, ascending stairs, descending stairs, sitting, standing, laying (6)	In the first trial, each subject placed the smartphone in a predetermined position i.e., the left side of the belt. However, in the second attempt, they could fix the phone in a desired position on the waist.
(2) [6]	4 (28–75 year) (45 ± 21.49)	ADXL335 accelerometer (connected to an ATmega328V microcontroller)	~8 Hz	waist, left thigh, right ankle, right arm (4)	walking, sitting, sitting down, standing, standing up (5)	The data have been collected during 8 h of five different activities for all subjects.
(3) [27]	8 (20–30 year)	accelerometer gyroscope magnetometer (Xsens MTx unit)	25 Hz	chest, right and left wrists, right side of the right knee, left side of the left knee (5)	walking in a parking lot, sitting, standing, lying, ascending/descending stairs, walking on a treadmill with a speed of 4 km/h (in flat and 15° inclined positions), etc. (19)	The subjects performed nineteen activities by their own style and were not controlled during data collection sessions.
(4) [33]	16 (19–83 year)	accelerometer (6-bit resolution)	32 Hz	right wrist (1)	walking, climbing stairs, descending stairs, laying down on bed, sitting down on chair, brushing teeth, eating meat, etc. (14)	There are postural transitions, reiterated and complex activities in the dataset.
(5) [34]	22 (25–35 year)	Accelerometer (Google Nexus One)	~30 Hz	jacket pocket on the chest (1)	walking (1)	The walking data of several subjects were collected in indoor and outdoor under real-life circumstances.
(6) [34]	15 (27–35 year)	accelerometer (Shimmer)	52 Hz	chest (1)	walking, walking and talking, standing, standing up, talking while standing, going up/down stairs, etc. (7)	They used a low-power, low-cost BeagleBoard with a Linux embedded operating system to transmit data over Bluetooth.
(7) [21]	17 (22–37 year)	accelerometer gyroscope magnetometer (Xsens MTx unit)	50 Hz	right and left calves, right and left thighs, back, right and left lower arms and right, left upper arms (9)	walking, jogging, running, jump up, rowing, cycling, etc. (33)	The dataset includes a wide range of physical activities (warm up, cool down and fitness exercises).
(8) [22]	10	accelerometer gyroscope magnetometer (Shimmer)	50 Hz	chest, right wrist, left ankle (3)	walking, sitting and relaxing, standing still, lying down, climbing stairs, running, cycling, etc. (12)	This dataset covers common activities of the daily living, given the diversity of body parts involved in each one, the intensity of the actions and their execution speed or dynamicity.
(9) [35]	14 (21–49 year) (30.1 ± 7.2)	accelerometer gyroscope (MotionNode)	100 Hz	front right hip (1)	walking forward, left and right, sitting and fidgeting, standing, going upstairs and downstairs, running forward, jumping up and down, etc. (12)	There were 5 trials for each activity and each subject performed the experiments on different days at indoor and outdoor places.
(10) [36]	20 (19–75 year)	accelerometer 2-axis gyroscope (attached to Tmote Sky)	30 Hz	waist, right and left wrists, right and left ankle (5)	walking forward, right-circle and left-circle, sitting, lying down, standing, going upstairs and downstairs, jogging, jumping, turning right and left etc. (13)	The design of the wearable sensor network was based on platform named DexterNet that implemented a 3-level architecture for controlling heterogeneous body sensors.
(11) [23]	4 (25–30 year)	accelerometer gyroscope (Samsung Galaxy S II)	50 Hz	belt, right arm, right wrist and right jeans pocket (4)	walking, sitting, standing, walking upstairs and downstairs, running (6)	Every participant performed each activity between 3 and 5 min. The smartphone was horizontally kept for belt and vertically for the arm, wrist, and pocket.
(12) [24]	36	accelerometer (Android-based smartphone)	20 Hz	front pants leg pocket (1)	walking, sitting, standing, upstairs, downstairs, jogging (6)	The android app, through a simple graphical user interface, permits to record the user’s name, start and stop the data collection, and label the activity being performed.
(13) [37]	19 (23–52 year)	accelerometer gyroscope magnetometer (Xsens MTx unit)	100 Hz	belt either on the right or the left part of the body, at the subject’s choice (1)	walking, sitting, standing, lying, running, falling, jumping (9)	Data were logged in indoor and outdoor settings under semi-naturalistic conditions.
(14) [25]	10 (25–30 year)	accelerometer gyroscope magnetometer (Samsung Galaxy S II)	50 Hz	right and left jeans pocket, belt position towards the right leg, right upper arm, right wrist (5)	walking, sitting, standing, walking upstairs and downstairs, jogging, biking (8)	All test protocols were carried inside a building, except biking.

**Table 3:** The accuracy and runtime of non-dominated classifiers.

Classifier ID	Accuracy (%)	Misclassification (%)	Runtime (ms)	Classifier ID	Accuracy (%)	Misclassification (%)	Runtime (ms)
**Waist**				**Left Upper Leg**			
21	93.82	6.18	9.31	21	96.69	3.31	3.52
28	94.02	5.98	9.57	24	96.62	3.38	3.39
108	93.75	6.25	9.24	57	95.93	4.07	**2.92**
109	94.07	5.93	10.41	60	96.11	3.89	3.11
183	93.75	6.25	8.92	222	96.15	3.85	3.16
189	93.72	6.28	**8.80**	267	97.63	2.37	34.73
190	94.04	5.96	10.13	268	97.86	2.14	102.90
267	95.48	4.52	45.95	269	**98.03**	**1.97**	151.83
268	95.51	4.49	121.58	**Right Lower Leg**			
269	**95.67**	**4.33**	196.10	16	**95.52**	**4.48**	113.05
**Right Lower Arm**				24	93.82	6.18	8.03
24	93.11	6.89	**7.61**	28	93.45	6.55	**8.02**
102	93.29	6.71	8.01	267	95.36	4.64	30.73
267	95.14	4.86	33.90	290	94.52	5.48	8.25
268	95.29	4.71	87.80	291	94.97	5.03	9.14
269	**95.30**	**4.70**	149.78	**Left Lower Leg**			
**Left Lower Arm**				21	94.26	5.74	7.91
28	92.29	7.71	7.59	25	93.16	6.84	**7.79**
57	91.50	8.50	**7.31**	267	95.99	4.01	32.36
63	91.61	8.39	7.35	268	**96.38**	**3.62**	83.45
102	92.24	7.76	7.50	290	93.64	6.36	7.89
267	**94.06**	**5.94**	32.50	291	95.02	4.98	8.85
**Right Upper Leg**				**Chest**			
24	97.93	2.07	7.04	21	96.17	3.83	6.97
57	97.33	2.67	**6.94**	84	95.25	4.75	**6.58**
63	97.43	2.57	**6.94**	87	95.43	4.57	6.95
183	98.05	1.95	7.49	105	96.48	3.52	7.63
189	97.97	2.03	7.21	168	95.39	4.61	6.91
267	**98.85**	**1.15**	32.12	183	96.37	3.63	7.02
291	98.14	1.86	8.08	267	97.52	2.48	29.64
				268	97.67	2.33	76.49
				269	**97.72**	**2.28**	125.10

**Figure A1:**
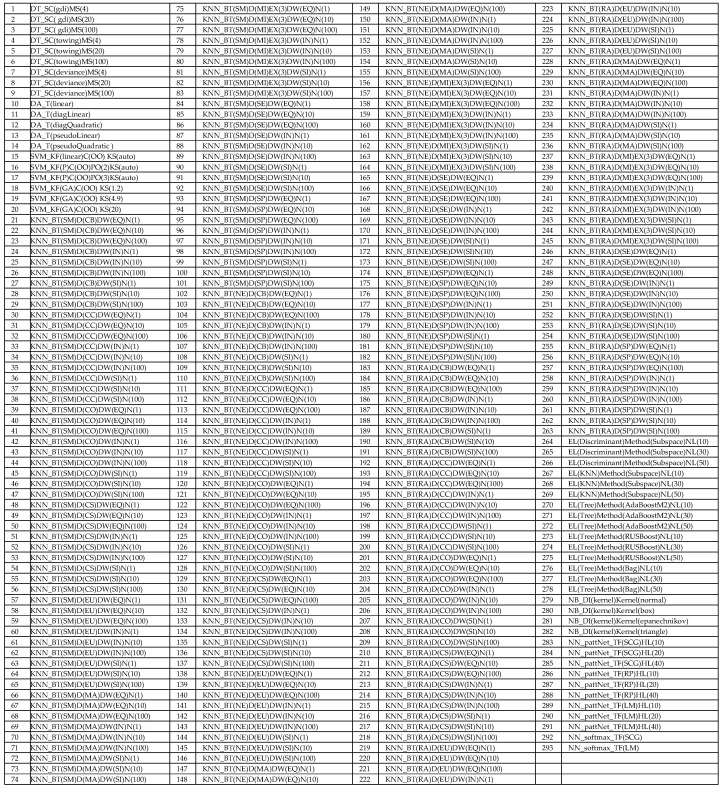
The list of the classifiers we explored in this paper. The numbers indicate the classifiers IDs.

## Abbreviations

The followings are the list of abbreviations: SC: Split Criterion; MS: Maximum number of Splits; T: Type; KF: Kernel Function; C: Coding; KS: Kernel Scale; P: Polynomial; GA: GAussian; OO: One-vs-One; PO: Polynomial Order; BT: Break Ties; D: Distance; DW: Distance Weight; EX: EXponent; N: number of Neighbors; SM: SMallest; NE: NEarest; RA: RAndom; CB: City Block; CC: ChebyChev; CO: COrrelation; CS: CoSine; EU: EUclidean; SE: Standardized Euclidean; MA: MAhalanobis; MI: MInkowski; SP: SPearman; EQ: EQual; IN: INverse; SI: SquaredInverse; EL: Ensemble Learner; NL: Number of Learners; DI: DIstribution; TF: Training Function; HL: number of Hidden Layers; SCG:: Scaled Conjugate Gradient; RP: Resilient backPropagation; LM: Levenberg–Marquardt backpropagation.

## Appendix A

In this section, the utilized approaches for activity classification problem are summarized while considering different parameters settings.

### Appendix A.1. Decision Tree

In this method, the discriminatory ability of the features is examined one at a time to create a set of rules. Decision Tree (DT) has been used in many studies [15,39], and the results show that it performs well with time and frequency domain features. In the top-down tree structure, each leaf represents a classification label and each branch denotes conjunctions of attributes that lead to the leaves. In other words, decision trees classify instances by starting at the root of the tree and moving through it (with decision being made at each node) until a leaf node. Generally, the utilized growing and pruning algorithms in decision tree induction are greedy and follow a recursive manner. The construction of a tree involves determining split criterion, stopping criterion and class assignment rule [40]. The most common techniques to measure the node impurity (for splitting nodes) are explained in Table A1 [41]. They define the node splits, where each split maximizes the decrease in impurity. In this study, we split branch nodes layer by layer until there are 4, 20 or 100 branch nodes. Therefore, we define nine different decision trees considering the mentioned split and stopping criteria. When a node is determined to be a leaf, it has to be given a class label. A commonly used class assignment function is the majority rule meaning that class *k* is assigned to node *t* as follows [42]:

$$l_{k}=\underset{i}{\max}p\left(i|t\right),t\in \tilde{T}$$

The set of terminal nodes (leafs) denoted by $\tilde{T}$.

### Appendix A.2. Discriminant Analysis

Discriminant Analysis (DA) is widely used in classification problems [43,44]. In this algorithm, there are three main elements: prior probability, posterior probability and cost [45]. A prior probability *P(k)* is the probability that an observation will fall into class *k* before you collect the data. There are two main choices, i.e., uniform and empirical. If the prior probability of class *k* is 1 over the total number of classes, it is called uniform. Besides, the number of training feature vectors of class *k* divided by all training features set defines the empirical prior probability.

**Table A1:** The common techniques for splitting nodes in DT.

Split Criterion	Description	Split Criterion	Description	Split Criterion	Description	
***Gini’s Diversity Index (GDI)***	$\sum_{i}p\left(i\right)\left(1-p\left(i\right)\right)=1-\sum_{i}p^{2}\left(i\right)$	***Deviance***	$-\sum_{i}p\left(i\right)log\;p\left(i\right)$	***Towing rule***	$p\left(L\right)p\left(R\right){\left(\sum_{i}\left|L\left(i\right)-R\left(i\right)\right|\right)}^{2}$	
					*Let L(i)/R(i) denote the fraction of members of class i in the left/right child node after a split and p(L)/p(R) are the fractions of observations that split to the left/right*	
	*p(i) is the probability that an arbitrary sample belongs to class li.*		*based the concept of entropy from information theory*			

A posterior probability is the probability of assigning observations to classes given the data. The product of the prior probability and the multivariate normal density explains the posterior probability that a point *x* belongs to label *k*. With mean $\mu_{k}$ and covariance $\Sigma_{k}$ at a point *x*, the density function of the multivariate normal can be described as below [45]:

$$\left(x|k\right)=\frac{1}{{\left(2\pi \left|\Sigma_{k}\right|\right)}^{1/2}}exp\left(-\frac{1}{2}{\left(x-\mu_{k}\right)}^{T}\sum_{k}^{-1}\left(x-\mu_{k}\right)\right)$$

The observed data *x* (the feature vector of one analysis segment) is classified to label *k* with the largest posterior probability. The posterior probability that an observation *x* belongs to label *k* is:

$$\hat{P}\left(k|x\right)=\frac{P\left(x|k\right)P\left(k\right)}{P\left(x\right)}$$

where *P(x)* indicates the probability of the feature vector *x* and is the sum over *k* of *P(x|k)P(k)*. Therefore, the predicted classification $\hat{y}$ with *m* classes is:

$$\hat{y}=arg\underset{y=1\ldots k}{min}\overset{m}{\underset{k=1}{\sum }}\hat{P}\left(k|x\right)C(y|k)$$

C(y|k) is the cost of classifying an observation *y* when its correct label is *k* [45]. In this paper, we consider five types of discriminant analysis classifiers: linear; and diagonal and pseudo variants of linear and quadratic types.

### Appendix A.3. Support Vector Machine

A Support Vector Machine (SVM) is defined based on one or a set of separating hyperplanes in a high dimensional space and was first proposed for binary-classification problems. SVM generates linear functions by considering a set of labels obtained from training dataset. The linear separator is created considering the maximum margin from the hyperplane to the support vectors [46,47]. By *n* training samples, $\left(x_{i},\;y_{i}\right);\;i\;=\;1,\;2,\;\ldots ,\;n$ we have:

$$\left\{x_{i},\;y_{i}\right\},\;i\;=1,\ldots n,\;y_{i}\in \left\{-1,+1\right\},\;x_{i}\in R^{d}$$

$y_{i}$ shows the binary nature of the classifier with either 1 or −1, representing the class of $x_{i}$. The $R^{d}$ is a d-dimensional vector space over the real numbers. The decision boundary of a linear SVM classifier is as follows:

$$w^{T}x+b=0$$

where *w* and *b* indicate a weight vector and bias, respectively. There are different linear separators, though SVM targets the one with maximum-margin hyperplane from any data point. The linear classifier is as follows:

$$f\left(x\right)=sign\left(w^{T}x+b\right)$$

The main goal is to find the best *w* and *b* which can maximize the geometric margin ($\frac{2}{\left|\left|w\right|\right|}$), with linear constraints $y_{i}(w^{T}x_{i}+b)\geq 1$ for all $\left(x_{i},\;y_{i}\right)$. This optimization problem can be defined as a minimization problem as follows:

$$\underset{w,b}{min}\left(\frac{1}{2}{\left|\left|w\right|\right|}^{2}\right)$$

$$s.t.y_{i}(w^{T}x_{i}+b)\geq 1,\;i=1,\ldots n$$

To solve this problem, the optimization function is transformed into the Lagrangian dual with the Karush–Kuhn–Tucker (KKT) conditions so that the Lagrange multiplier vector $\alpha_{i}$ is linked with each inequality of the constraints as:

$$\underset{\alpha \geq 0}{max}\underset{w,b}{min}\{\frac{1}{2}{\left|\left|w\right|\right|}^{2}-\overset{n}{\underset{i=1}{\sum }}\alpha_{i}[y_{i}(w^{T}x_{i}+b)-1]\},$$

$$\underset{\alpha \geq 0}{max}\;\left\{\overset{n}{\underset{i=1}{\sum }}\alpha_{i}-\frac{1}{2}\overset{n}{\underset{i=1}{\sum }}\overset{n}{\underset{j=1}{\sum }}\alpha_{i}\alpha_{j}y_{i}y_{j}x_{i}^{T}x_{j}\right\},\;s.t.\;\overset{n}{\underset{i=1}{\sum }}\alpha_{i}y_{i}=0$$

Thus, the optimal linear classification function is obtained as below, where $n_{sv}$ denotes the number of support vectors. Different studies show that SVM could provide an efficient non-linear classification and provide very promising results [48,49]. $K\left(x_{i},x_{j}\right)=\phi {\left(x_{i}\right)}^{T}\phi \left(x_{j}\right)$ is the kernel function which provides the inner product value of $x_{i}$ and $x_{j}$ in the feature space.

$$f\left(x\right)=sign\left(w^{T}x+b\right)=sign\left(\overset{n_{sv}}{\underset{i,j=1}{\sum }}\alpha_{i}y_{i}x_{i}^{T}x_{j}+b\right)$$

$$f\left(x\right)=sign\left(\overset{n_{sv}}{\underset{i,j=1}{\sum }}\alpha_{i}y_{i}K\left(x_{i},x_{j}\right)+b\right)$$

The most often used kernels in SVM are as described in Table A2. The type of the kernel function that transforms data from input space to a higher dimensional feature space has direct impact on the performance of the SVM classifier. Although there exist no well-defined rules for selecting the kernel type [46], here three well-known kernel functions are applied in SVM with Error Correcting Output Codes (ECOC) with One-Versus-One (OVO) technique to evaluate our multi-classification problem. ECOC breaks the multiclass task into a number of binary classification tasks which are then combined to output the final result [50]. The OVO coding design exhausts all combinations of class pair assignments. Therefore, if we have *K* distinct classes, the number of learners is $\frac{k\left(k-1\right)}{2}$. For each binary learner, one class is positive, another is negative, and the rest are ignored.

**Table A2:** The applied kernels in SVM.

Kernel	Formula	Kernel	Formula	Kernel	Formula
***Linear***	${x_{i}}^{T}x_{j}$	***Polynomial***	${\left({x_{i}}^{T}x_{j}+1\right)}^{d}$	***Radial basis function (RBF)***	$exp\left(-\frac{{\left|\left|x_{i}-x_{j}\right|\right|}^{2}}{2\sigma^{2}}\right)$

**Table A3:** The distance metrics in KNN.

Distance Metric	Description	Distance Metric	Description	Distance Metric	Description
***Euclidean***	$\sqrt{\sum_{i=1}^{n}{\left(x_{si}-y_{ti}\right)}^{2}}$	***Standardized Euclidean***	$\sqrt{\sum_{i=1}^{n}\frac{{\left(x_{si}-y_{ti}\right)}^{2}}{s_{i}^{2}}}$ $s_{i}$ *is the standard deviation of the* $x_{si}$ *and* $y_{ti}$ *over the sample set*	***Correlation***	$1-\frac{\left(x_{s}-\bar{x_{s}}\right){\left(y_{t}-\bar{y_{t}}\right)}^{\prime }}{\sqrt{\left(x_{s}-\bar{x_{s}}\right){\left(x_{s}-\bar{x_{s}}\right)}^{'}}\sqrt{\left(x_{s}-\bar{x_{s}}\right){\left(y_{t}-\bar{x_{s}}\right)}^{\prime }}}$ $\bar{x_{s}}=\frac{1}{n}\sum_{i}x_{si}$ *and* $\bar{y_{s}}=\frac{1}{n}\sum_{i}y_{ti}$
***City Block***	$\sum_{i=1}^{n}\left|x_{si}-y_{ti}\right|$	***Minkowski***	$\sqrt[{p}]{\sum_{i=1}^{n}{\left|x_{si}-y_{ti}\right|}^{p}}$ *In this work, p = 3*	***Mahalanobis***	$\sqrt{\left(x_{si}-y_{ti}\right)C^{-1}{\left(x_{si}-y_{ti}\right)}^{'}}$ *C is the covariance matrix*
***Chebychev***	$\underset{i}{max}\left\{\left|x_{si}-y_{ti}\right|\right\}$	***Cosine***	$1-\frac{x_{s}{y_{t}}^{\prime }}{\sqrt{\left(x_{s}{x_{s}}^{\prime }\right)\left(y_{t}{y_{t}}^{\prime }\right)}}$	***Spearman***	$1-\frac{\left(r_{s}-\bar{r_{s}}\right){\left(r_{t}-\bar{r_{t}}\right)}^{\prime }}{\sqrt{\left(r_{s}-\bar{r_{s}}\right){\left(r_{s}-\bar{r_{s}}\right)}^{\prime }}\sqrt{\left(r_{s}-\bar{r_{s}}\right){\left(r_{t}-\bar{r_{s}}\right)}^{\prime }}}$ $r_{si}$ *is the rank of* $x_{si}$ *over* $x_{s}.$ *If any* $x_{s}$ *values are tied, their average rank is computed*

### Appendix A.4. K-Nearest Neighbors

K-Nearest Neighbor (KNN) is based on a neighborhood majority voting scheme and assigns the new instance to the most common class amongst its *K* nearest. Simplicity and runtime are the main advantages of this method which used in several research works [15,26,51]. There are different metrics to determine the distance $d\left(x_{s},y_{t}\right)$ between two vectors $x_{s}$ and $y_{t}$. Table A3 describes the methods used in this study. The three applied distance weights are equal (no weighting), inverse (1*/d*) and squared inverse (1*/d*^2^). If multiple classes have the same smallest cost, the smallest index, the class with the nearest neighbor or a random tiebreaker among tied groups is used. Regarding selection of *k*, larger value may improve performance and reduce the effect of noise on the classification, but makes boundaries between classes are less distinct [52]. In addition, setting *k* to a too large value may destroy locality, and as a result, KNN looks at samples that are not neighbors. They are different techniques to select and find a good *k* [53,54]. Here, we consider three values 1, 10 and 100 for *k*; therefore, in total we run 243 KNNs with different settings.

### Appendix A.5. Ensemble Methods

Generally, ensemble classifier refers to a combination of different classifiers that are cooperatively trained on data set and then classify new data by taking a weighted vote of their predictions to obtain better predictive performance. Indeed, within the sensor-based recognition domain, different studies [6,15,20,25,26] report where an ensemble method outperformed a range of other classification models. Bagging, as named from the phrase “bootstrap aggregating”, is used to improve results of classification algorithms and help to avoid overfitting [55]. This ensemble method constructs bootstrap samples by repetitively resampling training instances with replacement. A sequence of classifiers $c_{1:b}$ (*b* = 10, 30, 50) in respect to variation of the training set is created by the bagging method. The prediction of a compound classifier, derived from the combinations of $c_{1:b}$, is given as:

$$c\left(d_{i}\right)=sign\left(\overset{b}{\underset{m=1}{\sum }}\alpha_{m}c_{m}\left(d_{i}\right)\right)$$

The above formula can be interpreted as to classify an example $d_{i}$ to the class with majority voting, and α should be chosen so that more accurate classifiers have stronger impact on the final prediction than less accurate classifiers [56]. More details about the theory of classifier voting can be found in [57].

Another approach is boosting which attempts to turn a weak leaner into a strong learner by gradually adapting how models are made. Each new model added to the ensemble is biased to take more notice to training instances that earlier models misclassified [45]. AdaBoost.M2 is a very prevalent boosting algorithm for multi-class classification. The algorithm trains learners sequentially and requires the weak learner to output an array of confidences associated with each possible labeling of an example. For every learner with index *t*, AdaBoost.M2 computes the weighted pseudo-loss for *N* observations and *k* classes as below [45]:

$$\varepsilon_{t}=\frac{1}{2}\overset{N}{\underset{n=1}{\sum }}\underset{k\neq y_{n}}{\sum }d_{n,k}^{\left(t\right)}\left(1-h_{t}\left(x_{n},y_{n}\right)+h_{t}\left(x_{n},k\right)\right)$$

where $h_{t}\left(x_{n},k\right)$ and $d_{n,k}^{\left(t\right)}$ are the confidence of prediction by learner and observation weights at step *t* for class *k*, respectively. The second sum is over all classes other than $y_{n}$ that is the true class. For more details, the reader is referred to [58].

RUSBoost is designed to improve the performance of models trained on skewed data. It combines data sampling and boosting, providing an effective method at classifying imbalanced data. It applies Random Under Sampling (RUS), a method that randomly takes out examples from the majority class for each weak learner in the ensemble until a preferred class distribution is found. If the smallest class has *N* training instances, classes with more instances are under sampled by taking only *N* observations. For reweighting and constructing the ensemble, it follows the procedure in AdaBoost.M2 [59].

Random subspace ensembles (Subspace) is similar to bagging except that the features are randomly sampled. Thus, subspace ensembles have the advantage of less memory and computations than ensembles with all features resulting in considerably shorter model training times. To train a weak learner, this technique selects a random set of *m* predictors (in this study, *m* = 12) from the *d* possible values without replacement. In our study, it repeats this procedure until there are 10, 30 or 50 weak learners. Finally, it takes an average of the score prediction of the weak learners, and classifies the observation with the maximum mean score [60].

In this manuscript, the boosting and bagging algorithms are based on tree learners, and the subspace has been applied to discriminant analysis and k-nearest neighbor learners.

### Appendix A.6. Naïve Bayes

Naïve Bayes (NB) is a powerful probabilistic classifier employing a simplified version of Bayes formula to decide on a class of a new instance [61]. In activity recognition, NB proved to perform well in the previous studies [20,62]. The following equation shows the Naïve Bayes under assumption of feature independence, though the assumption is usually violated in practice.

$$P\left(l|f_{1}\ldots f_{n}\right)=\frac{p\left(l\right)\prod_{i=1}^{n}p(f_{i}|l)}{p\left(f_{1}\ldots f_{n}\right)}$$

where *l* represents labels/classes (*l = 1, 2 …L*) and $f_{i}$ is a feature vector. The denominator of the right side of the equation is a constant, and p(l) is a prior. The posterior probability $P\left(l|f_{1}\ldots f_{n}\right)$ is determined by the likelihood $\prod_{i=1}^{n}p(f_{i}|l)$ and $p\left(f_{1}\ldots f_{n}\right)$ is the joint density of the predictors, so, $p\left(f_{1}\ldots f_{n}\right)=\sum_{l=1}^{L}p\left(l\right)\prod_{i=1}^{n}p(f_{i}|l)$.

**Table A4:** The applied kernel smoother types in NB.

Kernel Type	Formula	Kernel Type	Formula
**Uniform**	0.5 I{|x|≤1}	**Epanechnikov**	$0.75\;\left(1-x^{2}\right)\;I\left\{\left|x\right|\leq 1\right\}$
**Normal (Gaussian)**	$\frac{1}{\sqrt{2\pi }}e^{-0.5\;x^{2}}$	**Triangular**	(1−|x|) I{|x|≤1}

The Naïve Bayes classifier combines the independent feature mode with a decision rule. The common rule is known as the maximum a posteriori or MAP decision rule.

$$arg\;\underset{l}{max}\;P\left(l|f_{1}\ldots f_{n}\right)=arg\;\underset{l}{max}\prod_{i=1}^{n}p(f_{i}|l)$$

A typical assumption when dealing with data stream is that continuous values associated with each class are distributed according to a Normal (Gaussian) distribution. However, to alleviate this assumption, NB classifier computes a separate kernel density estimate for each class according to its training data [45]. There exists a large range of kernels that can be exploited for the kernel density estimate. Table A4 shows the kernel smoother types we applied in this study.

### Appendix A.7. Neural Network

Artificial Neural Network (NN) is generally presented as a system of interconnected neurons that are capable of machine learning. The basic processing unit of an NN is called perceptron and is a decision making unit with several inputs and a single output. The input neuron $p_{i}$ is weighted with an appropriate $w_{i}$. The perceptron sums the dot product of weights and inputs vectors, and adds a bias *b*. The obtained total signal will be transformed by a function which not only can be linear, but is most often a nonlinear transformation (e.g., log-sigmoid and tan-sigmoid) [63]. This process is summarized as:

$$a=f\left(b+\overset{i}{\underset{j=1}{\sum }}w_{j}p_{j}\right)$$

Feedforward neural networks are one of the most broadly used models in many real-world scientific problems. The network is divided into layers; therefore, it can learn nonlinear relationships between input and output vectors with nonlinear transfer functions. In the input layer, the nodes pass the values to the neurons or hidden units placed in the subsequent layer, which is called hidden layer. In this paper, we considered 10, 20, and 40 hidden neurons. The final layer is the output layer that depends upon the number of class labels in the classification problem [64].

In training the network, its parameters are adjusted incrementally until difference between the output units and the target values is minimized. Resilient backpropagation [65], scaled conjugate gradient [66] and Levenberg–Marquardt backpropagation are the most well-known network training algorithms. For example, Levenberg–Marquardt optimization uses the Hessian matrix approximation, $J^{T}J$, in the following Newton-like update [45]:

$$w_{i}\left(t+1\right)=w_{i}\left(t\right)-{\left[J^{T}J+\mu I\right]}^{-1}J^{T}e$$

where Jacobian matrix *J* holds the first derivatives of the network errors in respect of the weights and biases. *µ* stands for an adjustment factor, *e* for a vector of network errors and *I* for the identity matrix [63,67]. Resilient backpropagation network training algorithm updates weight and bias values according to the algorithm explained in [65]. In this method, the magnitude of the derivative has no influence on the weight update and only the sign of the derivative can define the direction of the weight update.

Therefore, in total, there are 293 different classifiers with different settings, as listed in Figure A1.
